# Adsorption of Zearalenone by *Aureobasidium pullulans* Autolyzed Biomass Preparation and Its Detoxification Properties in Cultures of *Saccharomyces cerevisiae* Yeast

**DOI:** 10.3390/toxins16020105

**Published:** 2024-02-15

**Authors:** Anna Bzducha-Wróbel, Monika Janowicz, Marcin Bryła, Iga Grzesiuk

**Affiliations:** 1 Department of Food Biotechnology and Microbiology, Institute of Food Sciences, Warsaw University of Life Sciences—SGGW, Nowoursynowska Str. 159c, 02-776 Warsaw, Poland; 2 Department of Food Engineering and Process Management, Institute of Food Sciences, Warsaw University of Life Sciences—SGGW, Nowoursynowska Str. 159c, 02-776 Warsaw, Poland; monika_janowicz@sggw.edu.pl; 3 Department of Food Safety and Chemical Analysis, Prof. Waclaw Dąbrowski Institute of Agricultural and Food Biotechnology—State Research Institute, Rakowiecka Str. 36, 02-532 Warsaw, Poland; marcin.bryla@ibprs.pl; 4 Department of Food Biotechnology and Microbiology, Institute of Food Sciences, Faculty of Food Technology, Warsaw University of Life Sciences—SGGW, Nowoursynowska Str. 159c, 02-776 Warsaw, Poland; grzesiuk.iga@gmail.com

**Keywords:** *Aureobasidium pullulans*, zearalenone, adsorption, detoxification, *Saccharomyce cerevisiae*

## Abstract

Different preventive strategies are needed to minimize the intake risks of mycotoxins, including zearalenone (ZEN). The aim of this study was to determine the ZEN adsorption ability of an autolyzed biomass preparation of polymorphic yeast *Aureobasidium pullulans* A.p.-3. The evaluation of the antitoxic properties of the preparation was also performed in relation to *Saccharomyces cerevisiae* yeast (ATCC 2366, ATCC 7090 and ATCC 9763) used as a model cell exposed to a toxic ZEN dose. The preparation at a dose of 5 mg/mL showed the adsorption of ZEN present in model systems at concentrations between 1 μg/mL to 100 μg/mL. The highest degree of adsorption was established for ZEN concentrations of 1 μg/mL and 5 μg/mL, becoming limited at higher doses of the toxin. Based on the Langmuir model of adsorption isotherms, the predicted maximum ZEN adsorption was approx. 190 µg/mL, regardless of pH. The growth of three strains of *S. cerevisiae* yeast cells in the medium with ZEN at concentrations within the range of 1.56 μg/mL–100 μg/mL was analyzed to determine the minimum inhibitory concentration. The growth of all tested strains was especially limited by high doses of ZEN, i.e., 50 and 100 μg/mL. The protective effect of the tested preparation was noted in relation to yeast cells exposed to toxic 100 μg/mL ZEN doses. The highest yeast cell growth (app. 36% percentage) was noted for a *S. cerevisiae* ATCC 9763 strain compared to the medium with ZEN but without preparation. More detailed tests determining the antitoxic mechanisms of the *A. pullulans* preparation are planned in the future, including cell culture bioassays and animal digestive tract models.

## 1. Introduction

According to the Annual 2022 Report of the Alert and Cooperation Network (RASFF, AAC and FFN, 2022) [1], mycotoxins are one of the most common contaminants identified in food and feed, threatening the health of consumers and animals. They are also the cause of economic losses in the food production chain. These substances have various directions and spectra of toxic effects on living organisms, e.g., zootoxic, phytotoxic and antimicrobial. Aflatoxin B1, deoxynivalenol, fumonisins, ochratoxin A, T-2 toxin and zearalenone (ZEN) are most frequently found in feed in the European Union [2].

Agricultural production, especially cereals, is very susceptible to mold contamination, often resulting in mycotoxin contamination. This problem affects approximately 25% of the world’s cereal crops [3]. ZEN is one of the most widespread mycotoxins in cereal, produced by molds of the genus *Fusarium* (e.g., *Fusarium graminearum*, *Fusarium cerealis*, *Fusarium culmorum*, *Fusarium equiseti*, *F. crookwellense* and *F. semitectum*). These fungi are responsible for the development of different plant diseases during the cultivation of cereal plants. ZEN contamination may also occur when grain is stored at a high humidity of 50% if the *Fusarium* infection occurs in the cultivation field during the pre-harvest period. The source of the discussed mycotoxin for humans and animals may be all species of small-grain cereals and corn, but also potatoes, tomatoes, exotic fruits such as papaya, legumes, pumpkin seeds, walnuts, vegetable oils and malt [4]. Contaminated cereal could be recognized as a significant source of ZEN and its masked forms in humans and animals because the majority of the average per capita caloric consumption and nutrients come from cereal and cereal-based products [4,5].

ZEN belongs to the group of non-steroidal mycoestrogens with high affinity for estrogen receptors ERα and ERβ. It is characterized by low acute toxicity, but its presence in the circulatory system results in functional changes in the reproductive system and animals’ pregnancy, and even in the development of breast, endometrial, testicular and prostate cancer or the stimulation of uterine fibrosis, cryptorchidism and hypospadias [6,7]. This compound may also have hepatotoxic, hematotoxic, immunotoxic and genotoxic effects, presumably by inhibiting the expression of tumor suppressor genes (PCDH11X, DKK1 and TC5313860) [8,9]. Pigs are most sensitive to the effects of ZEN. According to the EU Commission Recommendation (2006/576/EC), the recommended maximum zearalenone content in feed for piglets and gilts is 0.1 mg/kg, for breeding sows and boars in 0.25 mg/kg, and for ruminants is 0.5 mg/kg [10].

ZEN is a thermally stable compound; therefore, the processes used in the production of food and feed (e.g., thermal treatment, grinding) do not reduce the ZEN content [4,8,9]. European Union Commission Regulation 2023/915, concerned with the maximum levels for certain contaminants in food and repealing Regulation (EC) No. 1881/2006 [11], sets the maximum permissible levels of ZEN in foodstuffs, which range, depending on the product category, from 20 to 400 μg/kg of product. The European Food Safety Authority (EFSA) has indicated a tolerable daily intake (TDI) of 0.25 μg/kg body weight for the ZEN [12].

Different preventive strategies are still needed to minimize the intake risks of ZEN but also masked ZEN forms, e.g., mycotoxin decontamination in feedstuff. Mycotoxin decontamination or detoxification refers to methods of removing or neutralizing mycotoxins from the contaminated material [13]. One option is the use of adsorbents as feed additives, which can minimize the absorption of mycotoxins in the digestive tract of animals, therefore reducing the risk of harmful effects of these substances in the organism [14,15]. The application of non-nutritive mycotoxin-sequestering agents to feed or food is considered to be the most practical and effective way to mitigate the impact of mycotoxin contamination. Different mycotoxins adsorbents are described in the literature, including activated charcoal, hydrated sodium calcium aluminosilicate, montmorillonite, zeolite and different yeast cell origin preparations [13]. The exploitation of novel materials of adsorbents is an interesting direction in research. The polymorphic ‘yeast-like’ fungi *Aureobasidium pullulans* is a relatively well-studied fungal species recently proposed as bio-agent to control mycotoxin contamination and plant diseases [16,17]. The discussed species is also called “black yeast” due to the production of melanin. It is widely used in biotechnology, including the production of enzymes (e.g., amylase, xylanase, pectinase), single-cell proteins, and above all, pullulan, which is an extracellular polysaccharide with increasing use in the pharmaceutical, food and chemical industries [18]. *A. pullulans* itself is not currently included on the updated European qualified presumption of safety list (QPS) due to taxonomic inconsistencies [19]; however, the purified products of their biosynthesis (e.g., pullulan) are considered as safe. The discussed microorganism was included temporarily on the QPS list during recent years. It cannot be ruled out that, based on new scientific evidence, the clear recognition of *A. pullulans* as a safe microorganism will be possible in the future. This would open up possibilities for the practical use of the biomass of these fungi. Therefore, it is interesting to find a possible application for the waste biomass of these fungi after biotechnological processes. There are no published data available in the literature on the possible effect of the *A. pullulans* origin preparations on mycotoxin binding. Therefore, our research aimed to determine whether the autolyzed biomass of *A. pullulans* fungi, consisting mainly of cell wall polysaccharides, could be used in the ZEN adsorption process. Additionally, using a simple model involving the cultivation of three *S. cerevisiae* yeast strains in the presence of a toxic dose of ZEN, the potential protective effect of the preparation on the tested cells was determined. In our system, we treated *S. cerevisiae* cells as a simple model of a living eukaryotic organism exposed to an inhibitory dose of ZEN. Three randomly selected *S. cerevisiae* strains available at the American Type Culture Collection were used in this stage of the research. *S. cerevisiae* cells can be cultured easily, and their doubling time is short (approx. 2 h). They are extensively used as a model system for the wide type of eukaryotic cell biology studies (from cell cycle control to DNA damage response) [20]. They also do not raise ethical doubts compared to animal research. This is why we found this approach sufficient to initially illustrate the potential detoxification properties of the tested preparation in relation to *S. cerevisiae* cells.

## 2. Results

### 2.1. Chemical Composition of Autolyzed A. pullulans Biomass Preparation

The chemical composition of the obtained *A. pullulans* autolyzed biomass preparation is presented in Table 1. The preparation consisted mainly of sugar ingredients (approx. 62.5%), released after acidic hydrolysis of the preparation. It was characterized by a content of total glucans at the level of approx. 39%, including 37% of β-glucans and app. 1.9% of α-glucan. The protein content in the analyzed preparation was about 10%. The presence of 12 different amino acids was detected after hydrolysis of the tested preparation. The most abundant were histidine and arginine, which accounted for 22.2% and 16.7% of amino acids, respectively.

**Figure 1:**
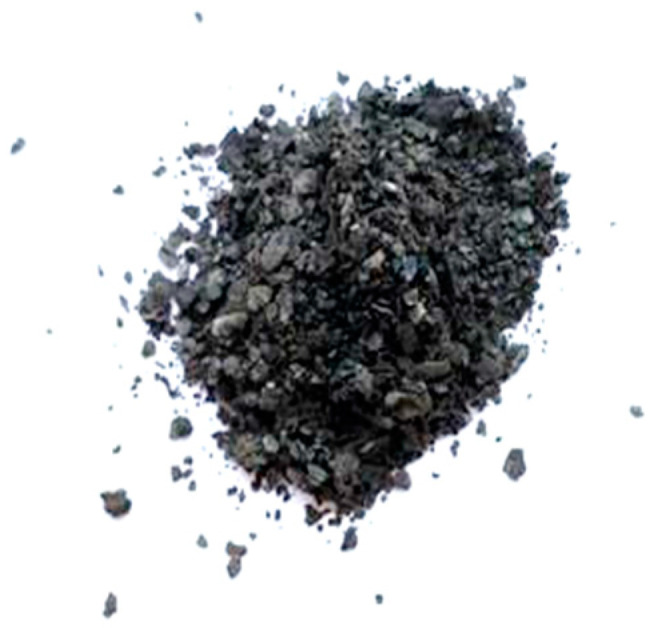
Macroscopic appearance of a lyophilized preparation of autolyzed *A. pullulans* biomass.

**Figure 2:**
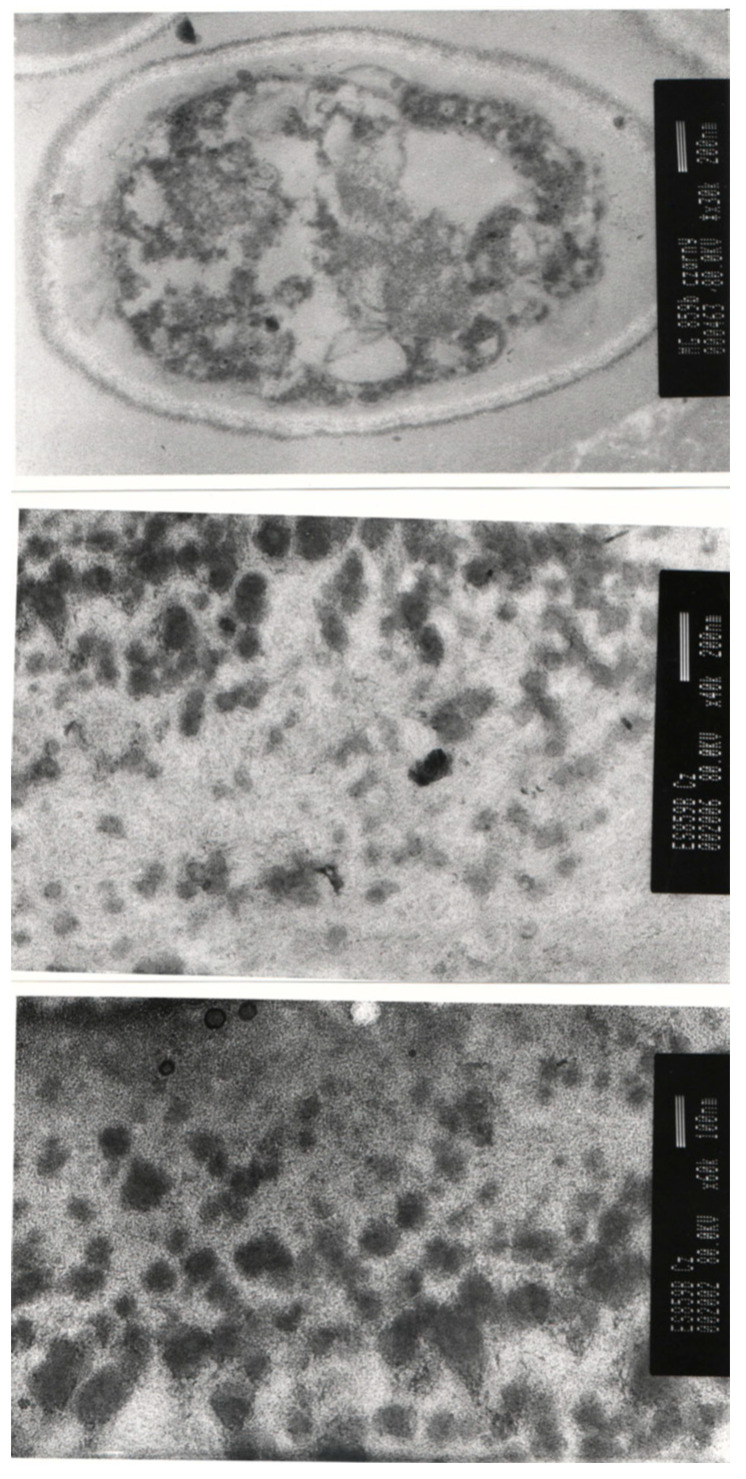
Electron microscopy visualization of *A. pullulans* cells (**a**) and melanin clusters in *A. pullulans* cell wall (**b**,**c**)—red arrows indicate melanin clusters in cell wall (photo taken and shared by M. Gniewosz according to the acknowledgments).

The studied *A. pullulans* autolyzed biomass preparation had a black color, which indicated the presence of melanin (Figure 1). Melanin is synthesized by *A. pullulans*, which is therefore called black yeast. These compounds are accumulated within the cell wall of non-conventional yeast, which is easily visible using electron microscopy (Figure 2).

### 2.2. Determination of ZEN Adsorption Efficiency and Adsorption Isotherm Using Autolyzed Aureobasidium pullulans Biomass Preparation as Binding Material

**Figure 3:**
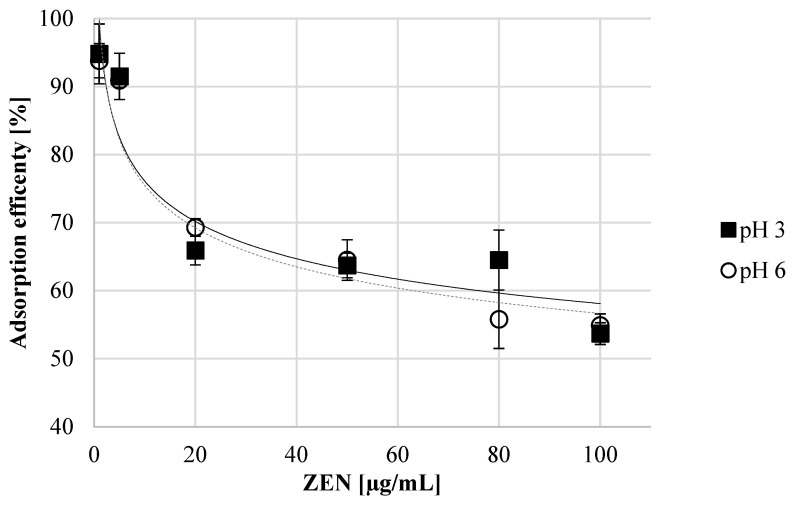
The ZEN adsorption efficiency at pH 3 and 6 of autolyzed *A. pullulans* biomass preparation as binding material.

The concentration of ZEN in the experimental system influenced the adsorption efficiency. As the concentration of ZEN increased, the mycotoxin binding efficiency of the tested *A. pullulans* A.p.-3 preparations (5 mg/mL) decreased. The discussed preparation had the highest binding efficiency in the presence of ZEN at concentrations of 1 and 5 µg/mL, respectively, at about 94% and 91%. At a ZEN concentration of 20–50 µg/mL, the preparation showed ZEN binding of approximately 63.7% and 69.3%, depending on the pH and ZEN concentration. An increase in the concentration of ZEN in the incubation system to 100 µg/mL resulted in a binding efficiency of approx. 53.7–54.9%, thus being similar at both pHs (Figure 3).

**Figure 4:**
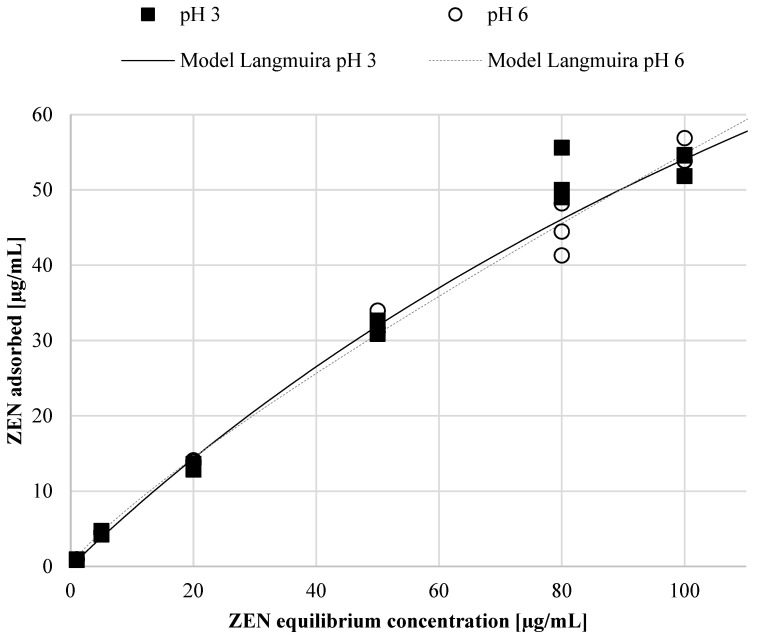
The optimal adsorption isotherms at pH 3 and 6 based on the Langmuir model.

The course of the adsorption isotherm determines the ability of the adsorbent to adsorb mycotoxin and describes the mechanism of the process. In order to describe the course of the obtained research results, three selected Langmuir, Freundlich and Hill models dedicated to the description of adsorption in the tested pH ranges were described. Based on the analysis of the obtained parameters of selected adsorption models, the experimental data were described using the Langmuir model, and the optimal ZEN fitting charts are shown on Figure 4.

For the tested experimental conditions, the Langmuir model showed a better fit than the Freundlich and Hill models, with an R^2^ (correlation coefficient) of 0.99; therefore, the Langmuir model was used to describe single-layer adsorption. It was assumed that adsorption occurred only in specific, localized places of the adsorbent, uniformly, and therefore adsorption was assumed to be homogeneous. It was also suggested that the K_L_ coefficients in the Langmuir model would be constant and indicate the stability of the bond. The analysis of the obtained model parameter values allowed for the observation of small K_L_ values of 0.0040 and 0.0042 depending on pH (Table 2), which indicates a strong connection between the toxin and the adsorbent. Another important factor of the Langmuir model is the R_L_ value, which indicates a favorable shape of the isotherm and the nature of the adsorption process (R_L_ > 1, unfavorable; R_L_ = 1, linear; 0 < R_L_ < 1, favorable; R_L_ = 0, irreversible). Furthermore, the predicted maximum adsorption amounts (Q max) were approx. 190 µg/mL at pH 3 and approximately 188 µg/mL at pH 6 (Table 2), corresponding to approx. 37.6–39.6 mg ZEN/per 1 g of the preparation.

### 2.3. Determination of the Minimal Inhibitory Concentration of Zearalenone in Relation to S. cerevisiae Yeast Cells

**Figure 5:**
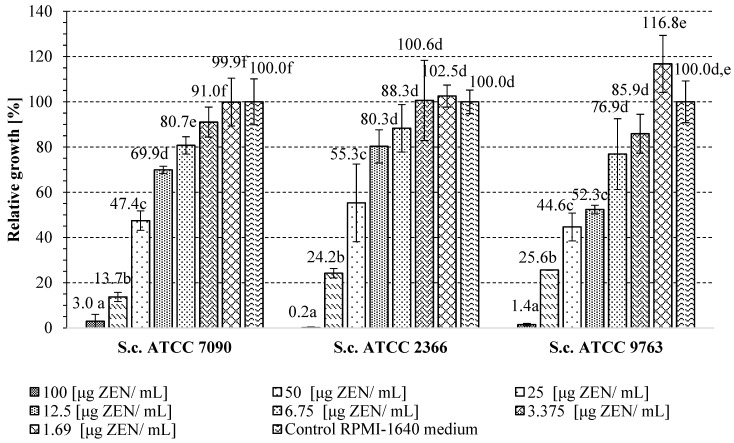
The effect of different doses of ZEN on the growth of studied *S. cerevisiae* yeast strains; a, b, c, …—mean values marked with the same letters do not differ significantly within results for each strain. Mean values were obtained based on results of at least 3 different cultures.

In this stage of this study, the influence of different doses of ZEN on the growth of three *S. cerevisiae* yeast strains (ATCC 2366, ATCC 7090 and ATCC 9763) was determined. In biological research, the yeast *S. cerevisiae*, with a well-known genome, is often used for model cells. The purpose is to indicate the dose of ZEN that inhibits studied yeast growth and use *Saccharomyces* cells as an example of a living organism for which the potential detoxification properties of the *A. pullulans* preparation with adsorption properties towards ZEN can be checked. This can be evaluated in the presence of a ZEN dose that inhibits the growth of the tested yeast. Therefore, the minimal inhibitory concentration was evaluated based on microdilution methodology and yeast growth in RPMI-1640 medium with the addition of 100; 50; 25; 12.5; 6.75; 3.13; and 1.56 ug of ZEN/mL. The obtained results are presented in Figure 5. The data showed the highest inhibition of the growth of the tested yeasts in the presence of 100 μg of ZEN/mL of culture, regardless of the *S. cerevisiae* strain. Therefore, this dose of toxin was selected for further research to determine the potential protective effect of the *A. pullulans* preparation on *S. cerevisiae* growth. In the case of the *S. cerevisiae* ATCC 9763 strain, the lowest dose of ZEN intensified the growth of the tested yeast, which may be related to the phenomenon of hormesis, i.e., the beneficial effect of small doses of harmful substances on the organism.

### 2.4. The Influence of A. pullulans Preparation on Yeast Growth in the Presence of Inhibitory ZEN Concentration

**Figure 6:**
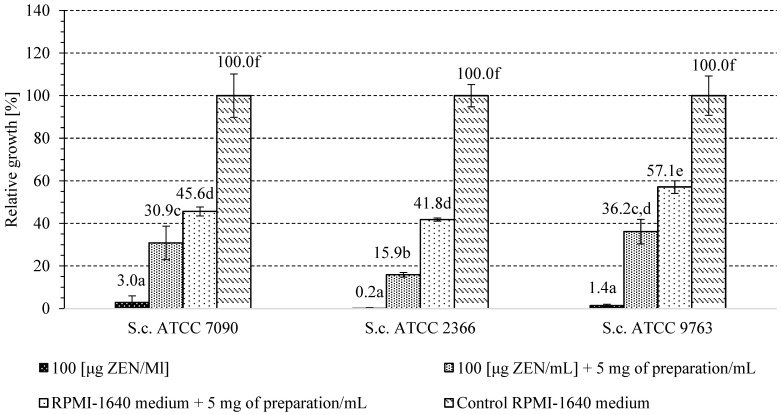
The effect of studied *A. pullulans* autolyzed biomass preparation on the growth of studied *S. cerevisiae* yeast strains in the presence of inhibitory ZEN concentration; a, b, c, …—mean values marked with the same letters do not differ significantly. Mean values were obtained based on results for 4 different cultures.

**Figure 7:**
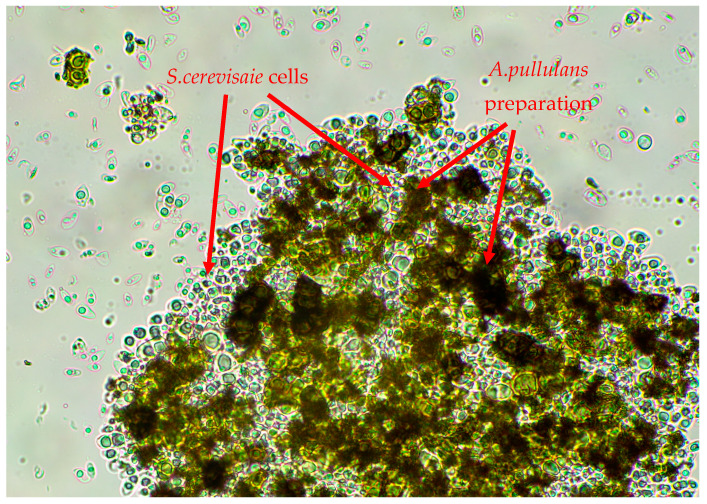
An example photo taken from a microscopic view of *S. cerevisiae* ATCC 7090 culture in RPMI-1640 medium with the addition of *A. pullulans* preparation (×600 magnification).

Based on the results of the previous stage of research, the dose of 100 μg/mL of ZEN, toxic to all tested strains of *S. cerevisiae* ATCC 7090, ATCC 2366 and ATCC 9763, was selected for further tests. The autolyzed *A. pullulans* biomass preparation rich in cell wall polymers was added at the concentration of 5 mg/mL to the culture media to determine its potential detoxification properties in relation to yeast cells. For each of the three strains tested, the addition of the studied preparation had a positive effect on cell growth in the presence of an inhibitory dose of ZEN (Figure 6). The highest growth was observed for the yeast *S. cerevisiae* ATCC 9763 and ATCC 7090 (approx. 30–36%), with inhibited growth in the culture with ZEN, but without the addition of the *A. pullulans* preparation. It can therefore be assumed that part of the toxin was bound by the preparation, contributing to the creation of a more favorable environment for *S. cerevisiae* cell growth. It should be noted that in the control samples containing a medium with the preparation inoculated with yeast cells, but without toxins, the growth of *S. cerevisiae* strains was less intensive compared to the culture carried out in a control RPMI-1640 medium. The growth was limited to approx. 41.8% for *S. cerevisiae* ATCC 2366, 45.5% for *S. cerevisiae* ATCC 7090 and 57.1% in case of *S. cerevisiae* ATCC 9763. Therefore, the preparation itself did not stimulate yeast multiplication but had detoxifying properties in medium with ZEN addition, probably by binding to part of the toxin, which allowed the yeast to activate protective pathways under lower ZEN concentrations in growth medium. Figure 7 shows an example photo of a microscopic preparation made from a culture of *S. cerevisiae* ATCC 7090 yeast in RPMI-1640 medium with the addition of *A. pullulans* autolyzed biomass. The *S. cerevisiae* yeast cells appear to be trapped within a particle aggregate of the *A. pullulans* preparation or to adhere to its surface. Perhaps the observed interaction between yeast cells and the *A. pullulans* preparation rich in melanin limited the growth of *S. cerevisiae* yeast.

## 3. Discussion

The biomass of yeast-like *A. pullulans* fungi contains approx. 30–35% of protein in the biomass dry substance, decreasing during the transition from the morphological stage with the shift from the form of blastospores to hyphae [21,22,23]. The protein content in studied autolyzed biomass preparation was lower because proteins were hydrolyzed under the influence of the activity of intracellular enzymes. The degradation products of proteins, but also polysaccharides, were to some extent lost during the washing steps of the production procedure, as it was observed for *S. cerevisiae* [24]. The obtained results indicate that the solid part of *A. pullulans* biomass remains after autolysis, consisting primarily of cell wall structural components, especially *β*-glucan polysaccharide. The ingredients were not easily available to yeast (polysaccharides and proteins) during *S. cerevisiae* cultivation in the presence of the preparation. Yeast cell wall proteins most often occur in the form of glycoproteins, mainly in its outer layer [25]. The mentioned cell wall polysaccharides and proteins are important for mycotoxin binding by yeast-based adsorbents [26]. The structural components of the *A. pullulans* cell wall are similar to those described for *S. cerevisiae*, which is the best known yeast in this regard [27,28]. There are literature data confirming the ability of *S. cerevisiae* preparations to bind mycotoxins when obtained, among others, via autolysis of cellular biomass [29]. However, there are no literature data describing mycotoxins adsorption capacity using *A. pullulans* preparations. Therefore, due to the innovative nature of the adsorbent obtained under our experiments, the discussion of the results was difficult and was mainly based on a comparison of our results with those described in the literature for *S. cerevisiae* preparations. The composition of fungi cell wall, the content of individual components and their structure may vary depending on the species, culture conditions and even the phase of the life cycle, which can influence the functional properties of discussed cellular components [30,31,32]. Schiavone et al. [33] showed that the process of *S. cerevisiae* (L71 and L69) cells autolysis and drying contributed to an increase in the roughness of the cell surface, which was observed as a change in the structure from relatively smooth to wrinkled. It can be assumed that the modification of the cell surface during autolysis and drying affects physicochemical properties, including the sorption capacity of the obtained material. It happens by increasing the contact surface and exposing it to functional groups capable of physicochemical interactions with other substances. Proteins and polysaccharides that make up the yeast cell wall participate directly in the binding of mycotoxins and other xenobiotics [34,35,36], thanks to the involvement of functional groups in intermolecular interactions, like –NH_2_, –COOH, –OH and –PO2. These occur in the components that build the wall structure. Non-covalent interactions occur, including hydrogen bonds, van der Waals forces and hydrophobic interactions. Cell walls contain carbohydrates (peptidoglycan, mannose, glucans), proteins and lipids, which may be involved in various adsorption mechanisms, e.g., through hydrogen bonds, or via ionic or hydrophobic interactions [37]. The relationship between *S. cerevisiae* cell diameter and cell wall thickness showed a correlation between wall surface area and ZEN removal capacity [15]. This proves that physical adsorption is in this case the main mechanism responsible for the removal of ZEN by yeast origin preparations. Yiannikouris et al. [38] studied the correlation between the content of *β*-D-glucan in *S. cerevisiae* cell walls and the mycotoxin binding capacity. They observed that *β*-D-glucan plays a major role in the adsorption of ZEA. The cited work used preparations of cell walls of 3 strains of *S. cerevisiae* with a glucan content of approximately 30–40% of the preparation, which is similar to the content of autolyzed *A. pullulans* biomass in our preparation. Yeast cell walls with higher glucan content were able to bind larger amounts of the toxin. The cited authors performed in vivo experiments to compare the ZEN binding capacity of yeast cell wall (YCW) extract and hydrated calcium aluminosilicate. It was found that YCW extract adsorbed ZEN more effectively in the gastrointestinal tract of monogastric animals. Moreover, it was able to adsorb 40% of the total ZEN content in the intestines [39]. However, no direct correlation between yeast composition (considering proteins, lipids, glucans, mannans and dry matter content) and mycotoxins adsorption capacity was found by Joannis-Cassan et al. [14], suggesting that complex phenomena of mycotoxin adsorption affect yeast-based products.

In recent years, the yeast *S. cerevisiae* has been investigated as a feed additive in production practice. Their effect as a detoxifying agent and in inhibiting the toxicity of ZEN was tested [40,41]. As already mentioned, several factors influence the efficiency of toxin adsorption. Autolyzed biomass does not only contain functional carbohydrates. The presence of protein and other ingredients in cell walls and significantly increasing the adsorption surface may result in more effective complexation of ZEN with purified β-glucan [42]. ZEN is considered as a non-polar compound that is adsorbed by hydrophobic surfaces. It has been proven that cell wall proteins and mannoproteins are related to the hydrophobicity of the yeast cell surface [43], which may vary based on amino acid composition.

Studies carried out using cell walls of *S. cerevisiae* strains RC008, RC009, RC012 and RC016 showed that, at the mycotoxin concentration of 1 µg/mL, the ZEN binding efficiency ranged from 48 to 87% [15]. It was therefore significantly lower at the indicated concentration compared to the results obtained in our studies. In the case of *Candida utilis* yeast cell wall preparations, ZEN was more efficiently bound by these preparations at a pH of 3.0, compared to pH 6.0 [42]. The use of *A. pullulans* autolyzed biomass allows the same binding capacity at different pH values. This means that the preparation would probably bind zearalenone with similar performance in different parts of the gastrointestinal tract, for instance, in the stomach (pH 3.0) and also in the small intestine (pH 6.0) in farm animals. It should be emphasized that the *A. pullulans* preparation was characterized by significantly better adsorption properties of ZEN compared to *S. cerevisiae* origin cell wall preparations. Decreases in the adsorption efficiency of ZEN by cell wall preparations of industrial brewing, bakery and distillery yeast were found with increases in the toxin concentration between 0.59 to 72.9 µg/mL [14]. This tendency is consistent with the observations of our work. However, the results of the cited work indicate that the adsorption of ZEN was a process reaching saturation, which was not observed under our research at even higher ZEN concentration.

*A. pullulans* is a polymorphic fungus with mycelium and chlamydospores, and is generally referred to as black yeast due to the formation of melanin pigment on its surface. It has been shown that melanin also has adsorbing properties. Currently, research in this area focuses on the binding of heavy metals, but this compound probably also affects the binding of mycotoxins [44]. In our experiment, the growth medium of *A. pullulans* was prepared according to Gniewosz et al. [45], who designed it to maximize the synthesis of pullulan by *A. pullulans*. In parallel to pullulan, other fungal metabolites are also produced, including cellular melanin. Large amounts of both substances accumulate in the cell wall of chlamydospores and in the shell formed during the maturation of these morphological forms [46]. The efficient binding of ZEN by the tested preparation of *A. pullulans* A.p.-3 could be due to the presence of melanin pigments in chlamydospores because our screening studies showed that the preparation obtained from the biomass of the *A. pullulans* B-1 strain, mutated to inhibit the synthesis of these pigments, bound less toxin (unpublished data). At the same time, it should also be noted that melanin has antimicrobial properties, including antifungal ones. This could be the reason for the observed reduction in the growth of the yeast *S. cerevisiae* in the presence of the *A. pullulans* preparation rich in this pigment [47].

Boeira et al. [48,49] analyzed the effect of ZEN on the growth of brewer’s yeast. The lower concentrations of ZEN used in the experiments (2 μg/mL and 25 μg/mL) had no effect on the growth of the tested strains. At a concentration of only 50 μg/mL after just 6 h of cultivation, we noted a significant reduction in the number of cells in the culture and their viability, as well as a decrease in the dry matter content. The toxic effect was stronger after 6 h of yeast multiplication compared to culturing after 24 h of incubation, which may indicate the existence of ZEN detoxification mechanisms in *S. cerevisiae* yeast cells. The impact of, among others, ZEN on the growth of 4 strains of brewer’s yeast in YPG medium was studied [50]. The presence of ZEN at a concentration of up to 20 μg/mL had no effect on the growth of the microorganisms used in the experiment. However, higher concentrations, like 50 and 100 μg/mL, inhibited the growth of biomass and growth rate. This effect had different intensities in individual strains, ranging from 50 to 80% compared to the control. It was observed that the yeast *S. cerevisiae* only developed a response to the presence of ZEN in the growth medium at the highest toxin concentrations, i.e., 50 and 100 μg/mL. However, this process was not observed in our study.

The phenomenon of hormesis, i.e., the beneficial effect of small doses of harmful substances on the body, has been known for a long time. This effect is explained by low doses of a toxin inducing minor oxidative stress, which stimulates the production of reactive oxygen species. Free radicals and peroxides, which act as signaling molecules, activate antioxidant processes and various metabolic pathways in cells, which in turn leads to accelerated growth. As it has been proven, the symptoms of oxidative stress are a frequently observed effect of mycotoxins on plant and animal cells [51,52]. The mentioned process was possible under a lower content of ZEN in yeast growth media in our experiments. In response to exposure to mycotoxins, yeast uses a universal cellular response pathway to stress factors, including stimulus perception, signal transduction and amplification, which leads to the induction or silencing of specific genes and, as a result, changes in cellular metabolism and other physiological processes. Transcriptome analysis of *S. cerevisiae* exposed to mycotoxin showed increased expression of genes involved in detoxification processes, antioxidant processes and DNA repair, which is also induced in response to oxidative stress [53]. In the case of *S. pombe* yeast treated with ZEN (500 μM), the inhibition of cell growth was accompanied by a decrease in glutathione content, an increase in the concentration of reactive oxygen species and the activity of antioxidant enzymes, a decrease in the content of sterols in the membranes, in particular squalene and ergosterol, and arrest of the cell cycle in the G2 phase [54]. In living cells, ZEN may undergo biotransformation processes leading to the formation of phase I or II metabolites. These are more polar compounds, more easily soluble in water, and therefore easier to remove and often much less toxic [9,55]. In yeast cells, including *S. cerevisiae*, a reduction in the carbonyl group results in the transformation of ZEN into α-zearalenol (α-ZOL) and *β*-zearalenol (*β*-ZOL), which are less harmful to cells, but α-zearalenol has more a potent estrogen-like effect than ZEN [9]. Presumably, the binding of some portion of ZEN in the culture medium by the adsorbent allowed yeast cells to activate detoxification mechanisms, which was reflected in cell growth in the presence of the *A. pullulans* preparation. More detailed and precise tests should be performed to determine the antitoxic mechanisms of the preparation in the future, including animals’ cell lines and digestive tract models. It is also important to indicate with what type of feed or food matrix the preparation could most effectively perform its detoxification function. Specific industrial application of the new preparation would require an assessment of its safety of use and an authorization procedure. Currently, an autolyzed *A. pullulans* biomass has no QPS qualification; therefore, it is not allowed to be used in animal feed.

## 4. Conclusions

The results of our research showed that the preparation of autolyzed *A. pullulans* biomass possessed adsorption properties towards ZEN at studied concentrations (between 1 μg/mL to 100 μg/mL), regardless of the pH of the experimental system. The highest degree of adsorption (approx. 94%) was noted for 1 μg of ZEN/mL. The process was limited to approx. 54% at the dose of 100 μg ZEN/mL. Based on the Langmuir model of adsorption isotherms, the predicted maximum ZEN binding was estimated at approx. 190 µg/mL, regardless of pH. The antitoxic properties of the studied preparation were demonstrated in the culture of three strains of *S. cerevisiae* propagated in the presence of an inhibitory dose of ZEN (100 μg/mL). The highest yeast cell growth (app. 36% percentage) was noted for the *S. cerevisiae* ATCC 9763 strain compared to the medium with ZEN, but without the preparation. It is advisable to carry out more detailed teststo determine the binding mechanism of the *A. pullulans* preparation to ZEN, but also other mycotoxins. The adsorption should be evaluated in systems imitating the digestive tract of animals or using cell culture bioassays. It is also interesting to determine which of the components of the *A. pullulans* preparation play the most important role in the process of mycotoxins binding.

## 5. Materials and Methods

### 5.1. Biological Material

The *Aureobasidium pullulans* A.p.-3 strain was used to obtain autolyzed biomass preparations. Three strains of *Saccharomyces cerevisiae* genus (ATCC 2366, ATCC 7090 and ATCC 9763) were used to study the influence of ZEN on yeast growth as a model cell. The presented microorganisms were obtained from the Pure Cultures Collection of the Department of Food Biotechnology and Microbiology, Institute of Food Sciences, Warsaw University of Life Sciences.

### 5.2. A. pullulans Cultivation and Autolyzed Biomass Preparation

The *A. pullulans* A.p.-3 non-conventional yeast cultivation was carried out in a medium for pullulan synthesis according to Gniewosz and Duszkiewicz [46]. The inoculum of *A. pullulans* was prepared in the medium mentioned above (50 mL in 500 mL flasks) by inoculation with cells collected from the slant. The culture was incubated at 28 °C for 72 h with shaking (180 rpm, SM-30 Edmund Buehler shaker). Cultures (50 mL of medium in 500 mL flasks) were started using 1 mL of inoculum. The biomass was collected after 96 h of cultivation at 28 °C/150 rpm via centrifugation in sterile falcons (5825× *g*/20 min, Eppendorf Centrifuge 580R, Eppnedorf AG, Hamburg, Germany). In the next step, the biomass was suspended in sterile distilled water and vortexed (Yellowline TTS2 vortex, IKA Works GmbH & Co. KG, Staufen, Germany). The suspension was re-centrifuged (parameters as above) and the supernatant was decanted again. The resulting biomass sludge was suspended in 150 mL of sterile 3% NaCl solution (pH = 5.0) to obtain a concentration of approx. 20% of biomass in the solution. Samples were placed in a water bath (Memmert WNB14, Memmert GmbH + Co. KG, Schwabach, Germany) at 55 °C for 72 h. After this time, methylene blue-stained microscope slides were prepared to check the viability of the cells. After the autolysis process was confirmed (blue-stained dead cells), the contents of the Schott bottles were transferred to sterile falcons and subjected to centrifugation (5825× *g*/5 min, Eppendorf Centrifuge 580R, Eppnedorf AG, Hamburg, Germany), followed by the decanting of the supernatant. Then, the sludge of the autolyzed biomass was washed with 30 mL of the following solutions in the order of their replacement: sterile distilled water, ethanol (96%) and water solution (2:1 by volume), 17 mmol NaCl, 34 mmol NaCl, 84 mmol NaCl, ethanol and water solution (2:1 by volume), and sterile distilled water [24]. With each solution, the falcon content was vortexed and then centrifuged (5825× *g*/5 min, Eppendorf Centrifuge 580R, Eppnedorf AG, Hamburg, Germany) to collect the autolyzed biomass. The autolyzed biomass was then freeze-dried using a Christ Freeze Dryer Alpha 1-4 LSC plus (Martin Christ Gefriertrocknungsanlagen GmbH P.O., Osterode am Harz, Germany), and used in adsorption tests.

### 5.3. Chemical Characterization of the A. pullulans Autolyzed Biomass Preparation

The content of total sugars, total glucans, *β*-glucan, α-glucan, and protein (all expressed as g/100 g) was analyzed, as was amino acid composition (expressed in %), to characterize the obtained preparation. The spectrophotometric method with 3,5-dinitrosalicylic acid (DNS) was used to evaluate the content of total sugars after hydrolysis with 72% H_2_SO_4_. The absorbance of the samples was measured at 540 nm against a blank sample (1 mL DNS and 1 mL distilled water) using a Rayleigh UV-1800 UV/VIS spectrophotometer (Beijing Rayleigh Analytical Instrument Co., Ltd., Beijing, China). The absorbance results were converted into the sugar content on the basis of the standard curve prepared for glucose. The content of total glucans, *β*-glucan and *α*-glucan was evaluated using the enzymatic *β*-Glucan Assay Kit (Yeast & Mushroom Assay Kit, K-YBGL, Megazyme, Bray, Ireland). The procedure was carried out according to the instructions of the producer. Calculations of glucans content were performed with the Mega-CalcTM automatic calculator available on the manufacturer’s website.

The protein content was determined via the Kjeldahl method. The preparation (app. 50 mg in replicates) was mineralized using 10 mL of 98% H_2_SO_4_ acid and Kjeltabs CT/3.5 as a catalyst. Mineralized samples were distilled in a KjelFlex K-360 apparatus (BÜCHI Labortechnik AG, Flawil, Switzerland). An automatic titration of the sample was performed using a TitroLine 5000 (SI Analytics GmbH, Mainz, Germany) and 0.1 mol/L HCl. The volume of HCl consumed was converted into nitrogen content and converted into protein using the factor 6.25 and expressed as g/100 g.

The determination of the amino acid composition in obtained preparations was performed using an AAA 500 automatic amino acid analyzer (Ingos s.r.o., Praha, Czech Republic), in which the separation was based on ion exchange chromatography. The methodology was based on the procedure described by Szkudzińska et al. [56].

### 5.4. Determination of ZEN Adsorption Isotherm Using Autolyzed A. pullulans Biomass Preparation as Binding Material

The first stage of this the research was to determine the pH stability of the buffer used in model systems of ZEN binding after adding the adsorbent. Changes in pH in the mycotoxin binding reaction environment may affect the adsorption efficiency, and so it is important to check how the dose of the preparation affects the pH of the experimental system. The test was performed in PBS buffer with pH 3.0 and pH 6.0, adding 5 mg/mL or 50 mg/mL of the tested *A. pullulans* preparation. The next step was to incubate the samples at 37 °C for 15 min. After this time, the pH of the samples was measured. The pH of the tested solutions changed in the case of a 50 mg adsorbent dose compared to a 5 mg dose, and hence a lower dose of the preparation was used in the toxin binding process. At the same time, the use of higher doses of the ZEN binder would likely hinder yeast growth to a greater extent, possibly preventing the detoxifying activity of the formulation from being observed. Moreover, reading yeast growth based on measurements of changes in the optical density of cells would be difficult in a medium with a high dose of the preparation.

The lyophilized preparation of autolyzed *A. pullulans* biomass was weighed into test tubes at a dose of 5 mg/mL, and PBS buffer (pH 3.0 or 6.0) prepared according to Bzducha-Wróbel et al. [42] was then added. Zearalenone solution was prepared by dissolving the mycotoxin (ZEN analytical standard, Romer Labs Diagnostics, Tulln, Austria) in acetonitrile and it was added to all tubes so that the final toxin concentration in the samples was 1 µg/mL, 5 µg/mL, 20 µg/mL, 50 µg/mL, 80 µg/mL and 100 µg/mL, respectively. For each pH variant and ZEN concentration, samples were prepared and analyzed in 5 replicates. Two types of negative control samples were prepared: PBS solutions (pH 3.0 and 6.0) with each ZEN concentration without the adsorbent, and PBS solutions (pH 3.0 and 6.0) with the adsorbent but without the mycotoxin. The next steps were to standardize the samples by vortex mixing, securing the tubes with parafilm and incubation (37 °C/90 min). After the incubation time, the tubes were centrifuged (14,100× *g* rpm/5 min, Mini SpinPlus centrifuge, Eppendorf AG, Germany) to separate the binder from the aqueous phase. Then, 900 µL of supernatant was collected into evaporative flasks with a volume of 5 mL. The flasks were stored at 4 °C until evaporation was carried out the same day. Evaporation was carried out using a vacuum evaporator (40 °C, 0 mbar). After evaporation, the samples were dissolved in phase (methanol/acetonitrile/water, 80:460:460). Then, the contents of the flasks were filtered using nylon syringe filters and 1.5 mL was taken into chromatography vials.

The determination of ZEN was carried out using a Knauer K 1001 high-performance liquid chromatograph (Knauer, Wissenschaftliche Geräte GmbH, Berlin, Germany) coupled with the RF-10AXL (Shimadzu, Kyoto, Japan) fluorescence detector. ZEN was separated on the Cosmosil 5C18-AR-II 4.6 μm × 250 mm (Nacalai Tesque, Kyoto, Japan) chromatographic column kept at a constant temperature of 45 °C. The separation was conducted in isocratic mode, in which the mobile phase consisted of a mixture of methanol/acetonitrile/water (80:460: 460, *v*/*v*/*v*) flowing at a rate of 1 mL/min. The total time for a single analysis was 35 min. The wavelengths corresponding to ZEN fluorescence emission and excitation were 274 and 446 nm, respectively. The retention time for ZEN was 15.18 min. The limit of detection of the method was 2 ng/mL (LOD—concentration of the given analyte at which its signal-to-noise ratio is 3:1). The limit of quantification was 7 ng/mL (LOQ—concentration of the given analyte at which its signal-to-noise ratio is 10:1). ZEN concentration was calculated based on the calibration curve. The amount of ZEN was calculated by subtracting the amount of free toxin found in supernatants after incubation in the presence of adsorbent from the amount of toxin found in the control samples without studied preparation (binder-free sample subjected to all steps of adsorption study). Preliminary studies confirmed that the quantities of ZEN in binder-free samples were the same as those in standard solutions of tested mycotoxin (without any pretreatment). This means that there was no degradation of mycotoxin during incubation in PBS buffer, no adsorption to used centrifuge tubes and no ZEN losses during sample preparation before analysis.

The ZEN adsorption efficiency (% ads) was calculated using the formula: %ads = (C_ads_/C_o_) × 100 = [(Co − C_aq_)/C_o_] * 100, where C_ads_—concentration of adsorbed ZEN (micrograms per milliliter), C_o_—concentration of ZEN in the supernatant of the blank control (with no adsorbent) (micrograms per milliliter), and C_eq_—the residual ZEN concentration in the solution at equilibrium (micrograms per milliliter). Based on the obtained results, three equations were tested to fit the data obtained for isotherm curves of Freundlich, Langmuir, and Hill according to Joannis-Cassan et al. [14].

### 5.5. Determination of the Minimal Inhibitory Concentration of ZEN in Relation to S. cerevisiae Yeast Cells

The inoculums of the studied *S. cerevisiae* yeast strains (ATCC 2366, ATCC 7090 and ATCC 9763) were prepared in aYPG medium (yeast extract—1%, peptone—2%, glucose—2%) in flasks with 90 mL of medium. The cultures were inoculated with yeast cells collected from the YPG slants. Yeast cultivations were carried out at 28 °C/180 rpm (reciprocating shaker SM-30 Edmund Buehler) for 18–19 h. The number of inoculum cells was determined by counting cells in the Thoma chamber to obtain approximately 10^5^ cells/mL initial cells number in the growth medium.

The RPMI-1640 medium, routinely used in research on the drug resistance of yeasts and molds, was used to determine the influence of ZEN on the growth of studied yeast *S. cerevisiae* strains. The minimal inhibitory concentration was evaluated based on the microdilution methodology. The yeast growth was tested in the presence of 100; 50; 25; 12.5; 6.75; 3.13; and 1.69 ug ZEN/mL. The yeast cultures were started by the application of 150 µL of RPMI medium with or without ZEN (control growth) and 10 µL of yeast cells inoculum (to obtain approximately 10^5^ cells/mL). The yeast cultures were grown at 28 °C for 48 h in 96-well microplates compatible with the Multiscan Sky reader (Thermo Fisher Scientific, Waltham, MA, USA). At least three replications for each culture for tested yeast strains were prepared (control medium and media with different ZEN concentrations). The yeast growth was determined on the basis of the optical density (OD) measurement at 600 nm on the Multiscan Sky apparatus (Thermo Fisher Scientific, Waltham, MA, USA) and referred to the control sample (RPMI-1640 medium inoculated with yeast without the addition of ZEN). The yeast growth in control medium was considered to be 100%. On this basis, the ZEN minimal inhibitory concentration was determined. A medium inoculated with yeast was also prepared with the addition of acetonitrile using the same volumes as those with which it was prepared for the ZEN solution used to obtain the desired toxin concentrations in the culture medium. The test was aimed at checking whether the acetonitrile present in the medium affects the development of yeast. The results obtained showed that there was no effect of the tested solvent.

### 5.6. Determination of the Effect of the A. pullulans Preparation on the Growth of Yeast in the Presence of an Inhibitory ZEN Concentration

The aim of this part of the research was to check whether the *A. pullulans* preparation could have a protective effect on living cells that were subjected to stress associated with inhibitory ZEN concentration in the growth environment. We used *S. cerevisiae* yeast cells as an example. For this stage of this study, the dose of 100 μg ZEN/mL that inhibited the growth of the tested yeast strains at a higher degree was selected. The preparation obtained from *A. pullulans* biomass (5 mg/mL) was added to 150 μL of RPMI medium with the addition of ZEN at the level mentioned above. In order to determine the effect of the preparation itself on the growth of yeast, there were also cultures prepared in which only the preparation was added to the RPMI medium along with yeast inoculum, but no toxin. The blank test was an RPMI medium with the addition of the preparation, but not one inoculated with yeast. Yeast growth was determined based on OD measurement at a wavelength of 600 nm in the MultiscanSky device (Thermo Fisher Scientific, Waltham, MA, USA) and was compared to the control sample (RPMI medium inoculated with yeast without the addition of the preparation), in which the growth was considered 100%. On this basis, we assessed whether the preparation added to the yeast culture could have a protective effect on it in the presence of an inhibitory dose of ZEN. Analyzes were performed for 4 parallel cultures of tested yeast strains propagated under the indicated media.

### 5.7. Data Analysis

Statistical data were evaluated using the STATISTICA V.13.1 software kit (StatPoint Technologies, Inc., Warrenton, VA, USA). Analysis of variance with the one-way ANOVA method and the HDS Tuckey test was carried out at the α = 0.05 level of significance to assess the significance of the differences.

## Figures and Tables

**Table 1:** Chemical composition of the autolyzed biomass preparation of *A. pullulans* A.p.-3.

Compound	Mass %
Total sugars	62.5 ± 1.3
Total glucans	38.9 ± 1.9
β(1,3)/(1,6)-glucan	37.0 ± 2.3
α-glucan	1.9 ± 0.2
Protein	9.6 ± 0.8
**Amino acids [mass % of total amino acids]**	
* Asp	6.6 ± 1.1
Thr	4.6 ± 0.9
Ser	4.3 ± 0.4
Glu	5.6 ± 1.1
Pro	6.4 ± 2.9
Gly	5.0 ± 0.1
Ala	6.0 ± 0.4
Val	6.2 ±0.8
Leu	7.5 ± 0.0
Tyr	6.9 ± 0.8
His	22.4 ± 2.4
Lys	4.0 ± 0.5
Arg	14.5 ± 1.6

* Description: Asp—aspartic acid; Thr—threonine; Ser—serine; Glu—glutamic acid; Pro—proline; Gly—glycine; Ala—alanine; Val—valine; Leu—leucine; His—histidine; Lys—lysine; Arg—arginine.

**Table 2:** Isotherm parameters of three models at pH 3 and 6.

Model	pH Conditions during Adsorption		Isotherm Parameters Depending on pH and Model		
Langmuir		Qmax	K_L_	R_L_	R^2^
	pH 3	189.9	0.0042	0.705	0.990
	pH 6	188.2	0.0040	0.712	0.997
Hill		Qmax	n_H_	R_L_	R^2^
	pH 3	109.7	1.228	-	0.981
	pH 6	199.1	0.971	-	0.988
Freundlich		Qmax	K_F_	n_F_	R^2^
	pH 3	-	1.232	1.202	0.988
	pH 6	-	1.236	1.215	0.998

## Data Availability

No new data were created or analyzed in this study.
